# Experiences of Preconception Counseling among Pregnant Women with Preexisting Diabetes: Opportunities to Improve Patient-Centered Care

**DOI:** 10.3390/ijerph20042908

**Published:** 2023-02-07

**Authors:** Cassondra J. Marshall, Lindsay Parham, Erin Hubbard, Roxanna A. Irani

**Affiliations:** 1 School of Public Health, University of California, 2121 Berkeley Way, Berkeley, CA 94720, USA; 2 Wallace Center for Maternal, Child & Adolescent Health, University of California, 2121 Berkeley Way, Berkeley, CA 94720, USA; 3 Division of Maternal-Fetal Medicine, Department of Obstetrics, Gynecology, and Reproductive Sciences, University of California, 490 Illinois St., San Francisco, CA 94143, USA

**Keywords:** diabetes mellitus, preconception care, maternal health, pregnancy, patient-centered care

## Abstract

Available research suggests that patients with diabetes do not regularly receive preconception counseling, but information on patients’ experiences of counseling is scant. We conducted a qualitative study involving semi-structured interviews with 22 patients between October 2020 and February 2021. Pregnant patients with preexisting diabetes were recruited from a specialty diabetes and pregnancy clinic at a large academic medical center in Northern California. Interviews were transcribed, coded, and analyzed using an inductive and deductive content analysis approach. A total of 27% reported they did not have any pregnancy-related discussions with a health care provider before pregnancy. Of those that did, many sought out counseling; this was often connected to how “planned” the pregnancy was. Few participants, nearly all with type 1 diabetes, reported having a formal preconception care visit. Participants described receiving information mostly about the risks associated with diabetes and pregnancy. While participants who sought out counseling generally reported their providers were supportive of their desire for pregnancy, there were a few exceptions, notably all among patients with type 2 diabetes. The varied experiences of participants indicate gaps in the delivery of pre-pregnancy counseling to patients with diabetes and suggest counseling may vary based on diabetes type. There are opportunities to improve the patient-centeredness of counseling.

## 1. Introduction

Diabetes is increasingly prevalent among women of reproductive age and during pregnancy [1,2,3]. The overall prevalence of diabetes among non-pregnant women of reproductive age in the United States (U.S.) is approximately 5% [3]; preexisting diabetes complicates approximately 1% of pregnancies [2]. Preexisting diabetes, including type 1 and type 2 diabetes, increases the risk of poor maternal, fetal, and neonatal health outcomes, including fetal anomalies, preeclampsia, fetal demise, preterm birth, and macrosomia [4]. Type 2 diabetes makes up the vast majority of diabetes cases among reproductive-aged women [1,5,6], and disproportionately impacts racial and ethnic minoritized and low-income populations. 

Preconception care—the counseling and care delivered prior to pregnancy—improves glycemic control prior to, and throughout, pregnancy, in order to reduce the risk of adverse outcomes. A recent systematic review and meta-analysis found that comprehensive pre-pregnancy care (both counseling and subsequent prenatal care) for patients with preexisting diabetes lowered hemoglobin A1c levels and reduced the risk of perinatal mortality, congenital malformations, preterm delivery, and neonatal intensive care unit admission [7]. For patients with reproductive capacity and preexisting diabetes, the American Diabetes Association recommends that routine diabetes care include preconception counseling, emphasizing the importance of pregnancy planning, appropriate glycemic control before pregnancy, and the use of effective contraception until pregnancy is safely desired.

Available research suggests that patients with diabetes do not regularly receive preconception counseling [8,9,10]. A recent study found that less than half of postpartum women with preexisting diabetes who had a recent live birth reported they received pre-pregnancy counseling [11]. While professional guidelines recommend counseling and studies have documented a need for improvements in the delivery of preconception counseling to individuals with diabetes, limited research has focused on patients’ experiences of counseling, particularly in the U.S. This information is critical to improving the development and delivery of patient-centered models of preconception counseling. Thus, the purpose of the present analysis is to explore and describe experiences of preconception counseling among patients with preexisting diabetes.

## 2. Materials and Methods

We used a qualitative approach to achieve our study aim, as it allows for in-depth exploration and the ability to provide both a comprehensive understanding of and rich descriptions of the complex phenomena of patient experiences in clinical settings [12]. The study was approved by the Institutional Review Boards of University of California, Berkeley, and the University of California, San Francisco. 

We used a purposive sampling strategy to recruit participants. Participants were recruited from a specialty diabetes and pregnancy clinic at a large academic medical center that serves over 20 counties in Northern California. To meet the study inclusion criteria, individuals needed to be currently pregnant, have a preexisting (i.e., prior to their current pregnancy) diagnosis of diabetes, be between the ages of 18 and 50, and have attended at least one medical appointment in the diabetes and pregnancy clinic, including the visit at the time of recruitment. Patients with gestational diabetes were excluded from the study. Interviews were available in English or Spanish; all interviews were conducted in English.

Participants were recruited by a review of clinic schedules to determine if there were any potentially eligible patients based on medical record data. Clinicians informed potentially eligible patients about the study, and a research coordinator contacted all interested participants and scheduled interviews. Interviews were conducted via telephone or video conference at the participants preference at a day and time that was convenient for the participant. REDCap software [13] was used for study management.

We developed a semi-structured interview guide based on a review of the relevant literature and the study team’s expertise. Two pilot interviews were conducted to refine the interview guide. The interviews broadly explored participants’ experiences of becoming pregnant, perspectives on pregnancy planning, attitudes, and beliefs regarding the impact of diabetes on their experience of becoming pregnant, and their health care experiences prior to their current pregnancy. The present analysis focuses specifically on patient experiences of preconception counseling.

Interviews were conducted between October 2020 and February 2021. Two clinical research coordinators conducted all interviews. Interviews lasted an average of 52 min, and participants received a USD 45 gift card incentive for their participation. Interviewers recorded detailed field notes after each interview and met with the study team throughout data collection to discuss the interview guide as well as data redundancy. 

All interviews were audio-recorded, professionally transcribed, and reviewed by a member of the research team for accuracy and clarity. We used a qualitative content analysis to analyze our interview data, using an integrated inductive and deductive approach. Our analytical process involved the following steps. First, two members of the study team reviewed all transcripts to familiarize themselves with the data and identify qualitative themes present in the data. Next, the study team developed a codebook with inductive codes based on this review of the data and deductive codes from the interview guide. Using the codebook, two members of the study team then coded four transcripts independently, writing analytic memos after coding each transcript, and discussing each coded transcript with each other to identify and resolve coding discrepancies. The study team then met to merge, refine, and discard codes as necessary, resulting in a finalized codebook. One member of the research team then coded all transcripts. The study team then met several times to identify salient themes by exploring code frequencies, code co-occurrences, mapping codes to higher-level categories, and memo writing. This step was completed for participants overall and by diabetes type (i.e., type 1 and type 2 diabetes). The study team used Dedoose qualitative data software [14] to manage and code all transcripts. Rigor and trustworthiness [15] were optimized by adhering to standards for qualitative research and enhanced by the creation of field notes and peer debriefing during data collection, double-checking transcribed interviews with audio recordings, independent coding by two individuals for a subset of transcripts, and use of analytic memos during analysis.

## 3. Results

The study sample included 22 participants. Table 1 presents diabetes type, participant sociodemographic characteristics, and pregnancy history for each participant. A total of 11 participants had a diagnosis of type 1 diabetes, 10 had a diagnosis of type 2 diabetes, and 1 had preexisting diabetes that was not classified as type 1 or type 2. Participants were 21 to 40 years in age (mean = 33.7 years) and were 8 to 32 weeks pregnant at the time of interview. Slightly over a third (36%) identified as white and 27% reported more than one racial/ethnic identity. Over 70% had private or employer-based health insurance; about two-thirds reported a college/bachelor’s degree or higher.

The majority of participants reported receiving some preconception counseling with respect to their diabetes before they became pregnant; over a quarter (27%) reported they did not have any pregnancy-related discussions with a health care provider before they became pregnant.

### 3.1. Theme 1: No Standard Version of Counseling

We observed no standard version of preconception counseling: when it did occur, it varied by provider type, type of clinical visit, and who initiated the conversation. Most commonly, participants received counseling from an endocrinologist, but some reported their obstetricians/gynecologists and primary care providers provided counseling. Counseling did not typically occur in a formal preconception care visit (*n* = 5), in which the purpose of the visit was solely for consultation about pregnancy with respect to diabetes. More commonly, counseling occurred in the context of other health care visits, often routine medical visits in which pregnancy was not the focus. Almost all of the participants who reported a preconception visit had type 1 diabetes (*n* = 4).

Counseling was often provided only when requested by patients. Of the participants who received counseling, many specifically sought out pregnancy-related information from their providers by mentioning their desire to become pregnant, either in the short or long term. In this way, preconception counseling was connected to how “planned” a pregnancy was. Table 1 offers descriptions, in the participants’ own words, of the nature of pregnancy with respect to planning.

*“I’m very glad that we had that opportunity, but I definitely had to seek it out. That [counseling] wasn’t necessarily going to be offered to me.”*

(Participant S)

Although uncommon, one participant described a contrasting example, in which their provider offered preconception counseling. The participant presented at a family planning clinic for a diabetes-related appointment and their provider prompted counseling by asking the participant if they had any plans on getting pregnant.

*“I went to a clinic regarding my diabetes and they asked me if I wanted to get pregnant. I told them ‘Yes’ ... and they told me that I should wait until I get my sugar under control.”*

(Participant T)

### 3.2. Theme 2: Information Provided during Counseling Typically Focuses on the Risk of Diabetes and Pregnancy

Patients who reported they had some preconception counseling described receiving information mostly about the risks associated with having preexisting diabetes during pregnancy. This referred to the medical complications that could arise during pregnancy due to poorly controlled diabetes, such as birth defects and miscarriage. We found that this was particularly true for those who had reported having a formal preconception care visit.

*“I felt like the focus was a lot [of] talking about the risks and all of the challenges.”*

(Participant N)

Some participants expressed the emotional impact of risk-based counseling, describing the counseling as causing worry, hopelessness, and devastation in one case. This participant reflected on hearing the risks associated with diabetes and pregnancy:

*“… they tell you, ‘Oh, you have high risk of literally everything. This is just a high-risk type situation.’ So naturally I was devastated because you never think about these things.”*

(Participant N)

### 3.3. Theme 3: Patients Seeking Counseling Felt Their Providers Were Generally Supportive of Their Pregnancy Desires, Although There Were a Few Exceptions

Most participants who sought out counseling felt their health care providers were supportive of their desire to become pregnant. They described providers offering resources to help patients achieve a healthy pregnancy, having positive, yet realistic attitudes about patients’ pregnancy goals, and clearly explaining information about managing blood sugar.

*“... they did a really good job of just explaining what it all meant and she was saying, ‘In my experience, you’re not going to have perfect blood sugars and that’s okay.’”*

(Participant K)

In a minority of cases (*n* = 3, all type 2 diabetes), participants described feeling unsupported about their desire for pregnancy. In one case, a participant described feeling “pushback” from their provider about their desire to become pregnant.

Notably, one participant shared that she had felt dissuaded from getting pregnant by her health care providers:

*“I also have heard that doctors … just tell me like, ‘Oh, yeah, you shouldn’t get pregnant while having diabetes.’ ‘Oh, yeah, it’s going to be a very high-risk pregnancy...’”*

(Participant L)

### 3.4. Theme 4: “Insider Knowledge”

A minority of participants (*n* = 5) who had worked in clinical care in some capacity shared that much of what they knew regarding diabetes and pregnancy was based on their professional, or “insider”, knowledge, which they then applied to their personal lives. This knowledge was not due to the clinical care they had received as a patient with diabetes. As such, they expressed that patients without a clinical background would be negatively impacted because they would likely not know enough to request information or resources from their health care provider as it relates to preexisting diabetes and pregnancy. 

One participant with type 1 diabetes asked:

*“I wonder, if I wasn’t a nurse, would I have known to bring it [pregnancy] up that far in advance? I don’t know.”*

(Participant P)

Another participant had a similar sentiment, noting:

*“The thing I struggle with … is not everyone knows what to ask at these things [preconception visits].”*

(Participant N)

### 3.5. Theme 5: A Desire for a Different Approach to Counseling

In addition to discussing their experiences with preconception counseling, participants shared their thoughts on how they believed counseling should be provided to patients. A key observation related to when and how pregnancy-related counseling occurs as part of ongoing diabetes care. There was a spectrum of desired approaches with respect to this. Some participants wanted information about pregnancy and diabetes offered as part of their routine diabetes care, before they wished to become pregnant. Other patients felt preconception counseling would not be particularly welcome if that did not match their current life stage (i.e., they were not trying to conceive). A participant who did not receive preconception counseling described feeling conflicted when asked if they would have wanted a health care provider to talk to them about pregnancy before they became pregnant:

*“I think looking back I would’ve liked somebody to say something. But I also don’t think that I would’ve been receptive to it.”*

(Participant G)

Other preferences about how counseling should be delivered were related to the nature and tone of the information provided. In one instance, a participant who received risk-based counseling (Theme 2) described wanting information that was less focused on the risks and adopted a more proactive stance that emphasized what could be done to help them achieve their pregnancy goals.

*“I think what I would have liked from that counseling would have been more of like, ‘If we work together as a medical team … this is what it looks like to have a healthy pregnancy with diabetes.’”*

(Participant N)

Another participant with type 2 diabetes emphasized the importance of health care providers being mindful of any bias:

*“I think that requires taking a moment and putting aside whatever preconceived notions you might have about the patient based on … age, race, what assumptions you might have of this person’s socioeconomic background.”*

(Participant J)

## 4. Discussion

The purpose of this qualitative inquiry was to better understand the experiences of preconception counseling among pregnant women with preexisting diabetes. We found that there was no standard experience of preconception counseling among participants in our study. The varied experiences of the participants are perhaps somewhat unsurprising given that, to our knowledge, there is no recommended, specific model of preconception counseling for adult patients with diabetes.

Several participants reported they did not experience preconception counseling with their health care providers before they became pregnant. Other studies, including those examining patient-reported counseling and those examining the documentation of counseling in health records, have found low rates of preconception counseling for patients with diabetes [8,10,16]. Similar to our study, Jazdarehee et al. also found that patients with diabetes frequently prompted counseling from their health care providers [17]. We observed that counseling was often connected to how planned a pregnancy was, meaning that a patient who desired pregnancy would present to their health care provider and ask for information on how their condition may impact pregnancy. Taken together, this suggests that there are likely gaps in counseling delivery for many patients, including those with pregnancies that are not planned and those who are less likely to raise the issue with their providers.

In our study, participants cited endocrinologists, obstetrician-gynecologists, and primary care providers as providing preconception counseling, with endocrinologists being the most common provider type. Other studies have found that provider type is a predictor of receipt of preconception counseling [10,16]. In one study examining contraceptive and preconception counseling among women with type 1 diabetes, endocrinologists and primary care providers had lower rates of documentation of counseling compared to maternal and fetal medicine specialists and obstetrician–gynecologists [16]. Future research should explore the role of provider type as an explanatory factor for our finding that there was no standardized version of preconception counseling for patients.

The findings of our study suggest experiences of preconception counseling may vary based on diabetes type. We found that formal preconception care visits in which preconception counseling was provided were uncommon for all participants, but nearly nonexistent among patients with type 2 diabetes. While, overall, participants felt supported by their providers regarding their desire for pregnancy, most instances in which patients felt unsupported were among patients with type 2 diabetes in our sample. Further, one participant raised issues of possible bias from providers. This is concerning with respect to patient-centered care and reproductive autonomy; additional research is needed to explore this finding in depth. Our findings suggest that interventions to improve the delivery of preconception counseling to patients with diabetes should consider tailoring approaches based on diabetes type. Type 1 and type 2 diabetes have differing etiology, clinical manifestation, and treatment [18]. Further, the two conditions are often managed by different providers. In their study of the frequency and content of preconception counseling among Canadian women with diabetes, Jazdarehee and colleagues found that women with type 1 diabetes were more likely to be managed by endocrinologists, while women with type 2 diabetes were more likely to be managed by their primary care providers [17].

Participants described ways that counseling could be improved and could better align with their preferences, thus offering suggestions for improvements in clinical care. Our findings suggest a need for a model of counseling that is proactive and balances information on the risks of diabetes and pregnancy with information on opportunities to optimize outcomes. Additionally, our findings suggest an approach to counseling that allows patients to tailor information and counseling based on their reproductive life stage and goals may be particularly welcome [19,20].

### Limitations

The strengths of our study include an in-depth exploration of the experiences and preferences of patients with both type 1 and type 2 diabetes, the racial and ethnic diversity of our sample, and our rigorous analytical approach. There are a number of limitations that should be considered. Our study population reflects that of a single-specialty diabetes and pregnancy clinic in one geographic location of the U.S. and, as such, does not represent the experiences of pregnant patients with diabetes more generally. We note that our sample was homogenous in terms of socioeconomic status; the majority of participants had private insurance and at least a college education. As described above, some participants had a background in clinical care. We also note that our exploratory study had a relatively small sample size and that, although we conducted our analyses by diabetes type, a deeper investigation is needed to assess possible differences in preconception counseling experiences by diabetes type. Another limitation is that participants were asked to recall and describe experiences that occurred before their pregnancy. It is possible that time and the experiences of their pregnancy may influence participants’ perception of conversations with their health care providers prior to their pregnancy. Finally, we were unable to explore how clinical factors, such as age at diagnosis, pre-pregnancy glycemic control, and specific medications taken, influenced patient experience of preconception counseling.

## 5. Conclusions

This study provides insight into previous research that has documented gaps in the delivery of preconception counseling to patients with diabetes. Our findings suggest the need for a routine pathway to receiving diabetes-specific preconception counseling, point to a need for a deeper exploration of the experiences of patients with type 2 diabetes, and offer suggestions for improving the patient-centeredness of counseling. To this end, this research suggests the need for a model of preconception counseling that does not solely focus on medical risks and complications, and that accounts for the possible emotional impact of risk-based conversations. Further, as our findings indicate there are varying perspectives as to when counseling should occur as part of ongoing patient care depending on reproductive goals, strategies that allow patients to tailor information provided in counseling based on their reproductive life stage should be considered. Finally, our findings point to a need to further investigate the role of clinician bias in diabetes-related preconception counseling, which can undermine the provision of patient-centered care.

## Figures and Tables

**Table 1:** Participant Characteristics.

Participant	Age (Years)	Race/ Ethnicity	Insurance	Education	Diabetes Type	Weeks Pregnant at Interview	Pregnancy History *	Person Pregnant with	Nature of Pregnancy
A	35	White	Private	College or more	1	32 weeks	Nulliparous	Husband	[The pregnancy was] “very planned”
B	39	White	Private	College or more	1	9 weeks	1 child	Husband	“…completely planned.”
C	40	Latina	Private	Some college	2	21 weeks	4 children, history of miscarriage, prior abortions	Fiancé	“I took a test just randomly and it was positive … I was not ready at all.”
D	32	White	Public and private	College or more	2	29 weeks	Nulliparous, prior abortion	Husband	“It was something that I had decided that I definitely wanted.”
E	35	Asian	Private	College or more	2	9 weeks	1 child, history of miscarriage	Husband	“We definitely discussed all the risks associated with getting pregnant at this time. And we decided that we’d give it a chance.”
F	33	White and Puerto Rican	Public	College or more	Preexisting diabetes not classified as type 1 or 2	8 months	Nulliparous	Husband	“I tried for many years to get pregnant naturally.”
G	32	White	Public and private	Some college	2	13 weeks, 3 days	1 child, history of stillbirth and fetal demise	Husband	“It wasn’t really a planned pregnancy. I had been diagnosed with diabetes type 2 for a couple of months just before this, and they were getting a hold on that, and then … I wanted to get pregnant, but I didn’t think that through.”
H	30	Algerian American	Private	College or more	1	8 weeks, 5 days	Nulliparous	Husband	“We were trying to get pregnant.”
I	35	Asian-Indian	Private	College or more	2	15 weeks	Nulliparous	Husband	“I was lucky to get pregnant in the first shot [referencing IUI].”
J	38	Black	Private	College or more	2	30–31 weeks	Nulliparous	Husband	“It was planned. I would say definitely planned.”
K	32	Hispanic	Private	College or more	1	21 weeks	Nulliparous	Husband	“It was very much planned.”
L	21	White and Latina	Public	Some college	1	28 weeks	Nulliparous, history of miscarriage	Boyfriend	“So, a lot of doctors have told me that there was very little, to no chance I’d get pregnant. So, I didn’t really expect it … I was shocked … I would say it was like a blessing in disguise.”
M	36	Japanese American	Private	College or more	2	About 9 weeks	Nulliparous, history of miscarriage	Husband	“I was open to it … I think because I had the miscarriage then for this pregnancy, I was much more happy and excited when we found out we were pregnant again.”
N	31	Hispanic, Asian, Black	Private	College or more	1	10 weeks	Nulliparous, history of miscarriage	Husband	“I was very much intentionally trying to get pregnant again … I was excited but cautious because I had had that miscarriage.”
O	34	White	Private	College or more	1	20 weeks	1 child, history of miscarriage	Husband	“We were not expecting to get pregnant as quickly … I mean, I’d say it’s planned because we really wanted a second child…”
P	33	White	Private	College or more	1	15 weeks	Nulliparous	Partner	“It was very much a planned one.”
Q	32	White	Public and private	Some college	1	26 weeks, 2 days	History of stillbirth	Husband	“We were both super happy and excited.”
R	38	White and Latina	Public and private	Some college	2	23 weeks	1 child, history of miscarriage	Husband	“Because of the pandemic, we weren’t sure … I mean, it was pretty planned in a sense that we did want a second one.”
S	32	White	Private	College or more	1	14 weeks	Nulliparous, history of miscarriage	Husband	“We were definitely actively trying.”
T	37	Mexican, Italian, Native American	Public	Some college	2	15 weeks	Nulliparous, prior abortion	Boyfriend	“It was a planned, I guess a planned surprise.”
U	35	Native American	Public	Some college	2	15 weeks	2 children, history of miscarriage	Husband	“It was [planned], but it was still kind of a surprise just because I wasn’t actually trying yet.”
V	32	White and Asian	Private	College or more	1	About 8 weeks	Nulliparous	Husband	“I wasn’t expecting it to just happen right away … I was very open, very excited with two positive tests.”

* Number of children does not include current pregnancy.

## Data Availability

The data are not publicly available due to the risk of deductive disclosure and sample size.

## References

[1] Healy A.M. (2022). Diabetes in Pregnancy: Preconception to Postpartum. Prim. Care Clin. Off. Pract..

[2] Alexopoulos A.-S., Blair R., Peters A.L. (2019). Management of preexisting diabetes in pregnancy: A Review. JAMA.

[3] Azeez O. (2019). Hypertension and Diabetes in Non-Pregnant Women of Reproductive Age in the United States. Prev. Chronic Dis..

[4] Association A.D. (2021). 14. Management of Diabetes in Pregnancy: Standards of Medical Care in Diabetes—2021. Diabetes Care.

[5] Simmons D. (2020). Paradigm Shifts in the Management of Diabetes in Pregnancy: The Importance of Type 2 Diabetes and Early Hyperglycemia in Pregnancy: The 2020 Norbert Freinkel Award Lecture. Diabetes Care.

[6] Bender W., Durnwald C. (2021). A Pragmatic Approach to the Treatment of Women With Type 2 Diabetes in Pregnancy. Clin. Obs. Gynecol..

[7] Wahabi H.A., Alzeidan R.A., Bawazeer G.A., Alansari L.A., Esmaeil S.A. (2010). Preconception care for diabetic women for improving maternal and fetal outcomes: A systematic review and meta-analysis. BMC Pregnancy Childbirth.

[8] Kachoria R., Oza-Frank R. (2014). Receipt of preconception care among women with prepregnancy and gestational diabetes. Diabet. Med..

[9] Marshall C., Britton L. (2019). Delivering family planning and preconception care to women with diabetes: Implementation challenges and promising strategies. Healthcare.

[10] Magdaleno A.L., Venkataraman S., Dion M., Rochon M., Perilli G., Vengrove M.A. (2020). Preconception Counseling in Women with Diabetes by Primary Care Providers and Perceived Barriers to Initiating this Discussion. Endocr. Pract..

[11] Marshall C.J., Huma Z., Deardorff J., Britton L.E. (2021). Prepregnancy Counseling Among U.S. Women With Diabetes and Hypertension, 2016–2018. Am. J. Prev. Med..

[12] Sofaer S. (1999). Qualitative methods: What are they and why use them?. Health Serv. Res..

[13] REDCap. https://www.project-redcap.org/.

[14] Home | Dedoose. https://www.dedoose.com/.

[15] Lincoln Y.S., Guba E.G. (1985). Naturalistic Inquiry.

[16] Disney E.A., Sanders J.N., Turok D.K., Gawron L.M. (2020). Preconception Counseling, Contraceptive Counseling, and Long-Acting Reversible Contraception Use in Women with Type I Diabetes: A Retrospective Cohort Study. Women’s Health Rep..

[17] Jazdarehee A., Shearer D., Thompson D., Lee J., Dahl M., Khurana R., Pawlowska M. (2021). The Power of Small Conversations: Bridging the Gap Between Diabetes and Pregnancy Planning. Can. J. Diabetes.

[18] Xu G., Liu B., Sun Y., Du Y., Snetselaar L.G., Hu F.B., Bao W. (2018). Prevalence of diagnosed type 1 and type 2 diabetes among US adults in 2016 and 2017: Population based study. BMJ.

[19] Callegari L.S., Aiken A.R.A., Dehlendorf C., Cason P., Borrero S. (2017). Addressing potential pitfalls of reproductive life planning with patient-centered counseling. Am. J. Obstet. Gynecol..

[20] Callegari L.S., Nelson K.M., Arterburn D.E., Dehlendorf C., Magnusson S.L., Benson S.K., Schwarz E.B., Borrero S. (2021). Development and Pilot Testing of a Patient-Centered Web-Based Reproductive Decision Support Tool for Primary Care. J. Gen. Intern. Med..

